# Exploring Quantum Dots Size Impact at Phase Diagram and Electrooptical Properties in 8CB Liquid Crystal Soft-Nanocomposites

**DOI:** 10.3390/nano13222980

**Published:** 2023-11-20

**Authors:** Stefanos Basim Atata, Ioannis Lelidis

**Affiliations:** Faculty of Physics, National and Kapodistrian University of Athens, Panepistimiopolis, Zografos, 15784 Athens, Greece

**Keywords:** anchoring energy, birefringence, Fréedericksz transition, liquid crystals, quantum dots, soft-nanocomposites, switching times

## Abstract

We explore the influence of functionalized core–shell CdSe/ZnS quantum dots on the properties of the host liquid crystal compound 4-cyano-4′-octylbiphenyl (8CB) through electrooptical measurements. Two different diameters of quantum dots are used to investigate the size effects. We assess both the dispersion quality of the nanoparticles within the mixtures and the phase stability of the resulting anisotropic soft nanocomposites using polarizing optical microscopy. The temperature-mass fraction phase diagrams of the nanocomposites reveal deviations from the linear behavior in the phase stability lines. We measure the birefringence, the threshold voltage of the Fréedericksz transition, and the electrooptic switching times of the nanocomposite systems in planar cell geometry as functions of temperature, mass fraction, and diameter of the quantum dots. Beyond a critical mass fraction of the dopant nanoparticles, the nematic order is strongly reduced. Furthermore, we investigate the impact of the nanoparticle size and mass fraction on the viscoelastic coefficient. The anchoring energy at the interfaces of the liquid crystal with the cell and the quantum dots is estimated.

## 1. Introduction

Liquid crystals (LCs) or mesophases combine fluidity and crystal anisotropy [1,2,3]. LCs-based devices are ubiquitous [4,5,6,7,8,9]. Nanoparticles (NPs) exhibit unique properties and behaviors due to their small size that implies a high surface area-to-volume ratio. These properties make them valuable in a wide range of applications [10,11,12,13]. When nanoparticles are introduced in a liquid crystalline matrix, they can strongly affect the properties of the host LC and consequently impact the performance of devices. The versatility of LCs doped with NPs makes them valuable for a wide range of applications in optics, electronics, biomedicine, energy, and beyond. It has been observed that the properties of the LC-host are primarily affected by the geometry of the NPs (size, shape), their bulk and/or surface properties, and their concentration. Over the past two decades, numerous theoretical and experimental studies have been appeared regarding the influence of NPs on phase transitions, orientational order, and the response of the host liquid crystalline matrix to external fields [14,15,16,17,18,19,20,21,22,23,24,25,26,27,28,29]. Naturally, the well-known Fréedericksz transition has been studied too in certain soft-nanocomposite materials for the voltage threshold of this transition is crucial for applications.

Quantum dots (QDs) have been extensively investigated due to their applications in photovoltaics, solar cells, biomedicine, lasers, photonics, liquid crystals, and more. When doping an LC with QDs, one can tune various properties of the host such as capacitance, electrical conductivity, dielectric properties, ion trapping, elastic constants, electrooptical properties, viscosity, topological defects, alignment of the nematic director, and phase thermodynamics [30,31,32,33,34,35,36,37,38,39]. It is well known that the electronic and optical properties of QDs critically depend on their size [40]. In what concerns the doping of LCs with QDs, understanding the interaction between LCs and QDs is a prerequisite for developing new nanocomposite materials.

A few years ago, we conducted a study on the impact of CdSe/ZnS core–shell QDs on the nematic order of the LC compound 4-n-pentyloxyphenyl-4′-n-octyloxybenzoate (5OO8) [41,42]. It was evidenced that the NPs can reduce the orientational order parameter. Beyond a certain concentration threshold, the nematic phase is disrupted, leading to the emergence of a “paranematic” phase. Recently, similar investigations have been published for the liquid crystalline compound 8CB doped with QDs both in the nematic and smectic-A phases [43]. Since QDs locally promote homeotropic alignment around them, the local director deviates from the global nematic orientation in a uniformly aligned cell. Nevertheless, for low concentrations, the global nematic orientation remains well-defined, possibly indicating that the local director distribution, on the average, aligns with the direction of the cell.

In the current paper, we focus on the impact of QDs’ size in mixtures with the liquid crystalline compound 8CB on both the phase diagram and various electrooptical properties of the system. Specifically, we explore, as a function of the QDs’ size and mass fraction, the orientational order, clearing temperature and the temperature range of the nematic phase. A large drop of the clearing temperature was measured alongside a narrowing of the nematic phase temperature window. Additionally, we delve into the Fréedericksz transition threshold and the viscoelastic properties in a splay geometry cell. The rotational viscosity coefficient which relates to the reorientation process of the nematic director is of utmost importance as it governs the switching speed of devices. In particular, it enters in the switching times via the viscoelastic coefficient of the nematic phase. The anchoring energy on the LC–QD and LC–cell interfaces is calculated/measured. Our experimental approach employs standard electrooptical methods tailored for birefringent materials.

The paper is organized as follows: In Section 2, we detail the materials and experimental methods employed. Section 3 is dedicated to presenting the experimental results on soft-nanocomposites including microstructure, phase diagrams, orientational order parameter, orientational transition of the nematic director, switching times, and viscoelastic coefficient. In Section 4, we discuss anchoring energy at the cell–LC and the QD–LC interfaces. Finally, in Section 5, we engage a discussion of the results and draw conclusions.

## 2. Materials and Methods

The investigated LC compound 8CB was procured from Frinton Laboratories and used without further purification. When cooling from the isotropic (I) phase, 8CB exhibits nematic (N) and smectic-A (SmA) phases at temperatures of 313.65 K and 306.65 K, respectively. Its molecule possess a strong dipole moment of ≈6 D nearly parallel to the long molecular axis, giving rise to a strong positive dielectric anisotropy. The physical properties of 8CB have been extensively studied in existing literature. Detailed data are available for elastic constants, refractive indices, viscosity, order parameter, dielectric constants, etc. The core–shell hydrophobic QDs consist of a CdSe core capped with a ZnS shell and were procured from PlasmaChem. According to PlasmaChem, the ZnS shell has a thickness of 0.6±0.1 nm. These QDs are functionalized with trioctylphosphine oxide (TOPO). For our investigations, QDs with core diameters of 2.5 nm and 6.7 nm were used. The total diameters of the QDs, including the surfactant layer, are approximately $D_{t}$ = 5.1 nm for the tiny ones and $D_{\ell }$ = 9.3 nm for the large ones. Several 8CB-QDs mixtures were prepared using the following protocol: Predetermined quantities of 8CB and QDs were separately dispersed in toluene, with both solutions undergoing 2 h of sonication. Required volumes of the solutions were combined to create each 8CB–QD mixture, followed by 9 more hours of sonication. The solvent was then evaporated overnight under magnetic stirring on a heated stage. Mixtures with various mass fractions $\chi =\frac{m_{qd}}{m_{qd}+m_{lc}}$, where $m_{qd}$ is the mass of QDs and $m_{lc}$ is the mass of the LC-compound, were prepared including χ = 0.13%, 0.25%, 0.4%, 0.5%, and 0.6% per weight, hereafter called $M_{i}^{t}$, where the superscript denotes the size of the QDs (*t* for core diameter of 2.5 nm) and the subscript *i* = 1–5 denotes the increasing χ value. Correspondingly, for QDs with core diameter of 6.7 nm, the mass fractions of the prepared mixtures were 0.03%, 0.12%, 0.24%, 0.48%, and 2.4% per weight, hereafter called $M_{i}^{\ell }$, where *i* = 1–5. Hereafter, we call the NC with the large (tiny) QDs as the *ℓ*-type (t-type) system. Each mixture was introduced, by capillary suction, into a planar cell with a gap, *d*, of 5μm at a temperature above the clearing temperature $T_{IN}$. The cell is of antiparallel geometry, and in the absence of an external field, the LC exhibited a uniform homogeneous orientation. The pretilt at the interfaces is $\approx 3^{\circ }$. A Leica DM2500P polarizing optical microscope (POM) was used for optical observations. The POM is equipped with a Leica DFC420 digital image acquisition camera controlled by a PC. The temperature was regulated by an Instec HCS402 heating-stage that was mounted on the microscope circular stage. The temperature stability was better than 100 mK.

Birefringence measurements, Δn, were conducted using a Berek compensator and/or an optical method [44,45] described in the following paragraph. The scalar orientational order parameter, *S*, was deduced from $S=\Delta n/\Delta n_{0}$[1,3], where $\Delta n_{0}$ represents the birefringence associated with perfect nematic order (S=1).

### Electrooptical Measurements Setup

**Figure 1:**
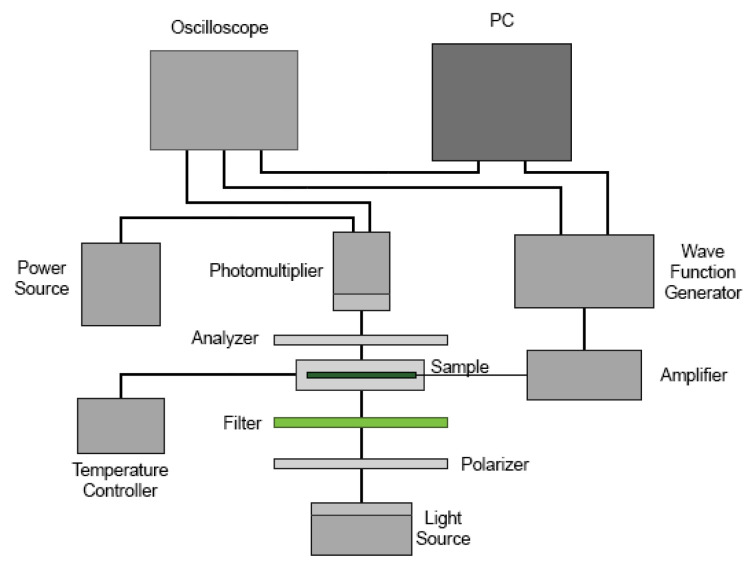
Schematic diagram of the experimental setup for the electrooptical measurements.

The electrooptical response of the sample was measured using the experimental setup outlined in Figure 1. The setup comprises a POM equipped with a photomultiplier tube (Hamamatsu Photonics K.K., H10721, Shizuoka, Japan) which is connected to an oscilloscope (Tektronix, Beaverton, OR, USA, model TDS 2024C). A waveform generator (Keithley Instruments, Inc., Cleveland, OH, USA, model 3390) and a voltage amplifier (FLC Electronics, Partille, Sweden, model A400) were employed to apply an external electric field to the LC–cell. The overall configuration is managed by a PC. The incident light’s wavelength was fixed at λ=546.3 nm through an interference filter. Both the applied electric potential and the resulting response signal were digitally recorded for subsequent analysis.

**Figure 2:**
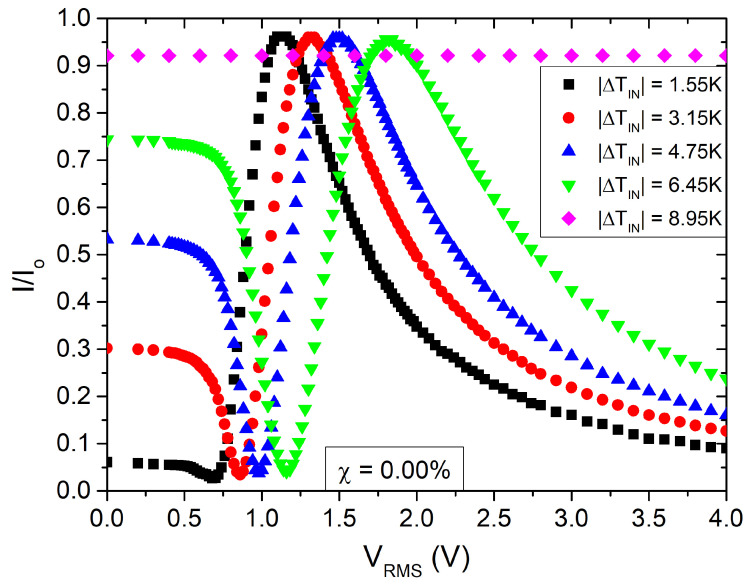
Normalized transmitted light intensity $I/I_{0}$ vs. the applied AC voltage amplitude $V_{rms}$ for a planar cell of pure 8CB. Rubbing direction at π/4 to P⊥A. Several isotherms are shown. *f* = 1000 Hz.

**Figure 3:**
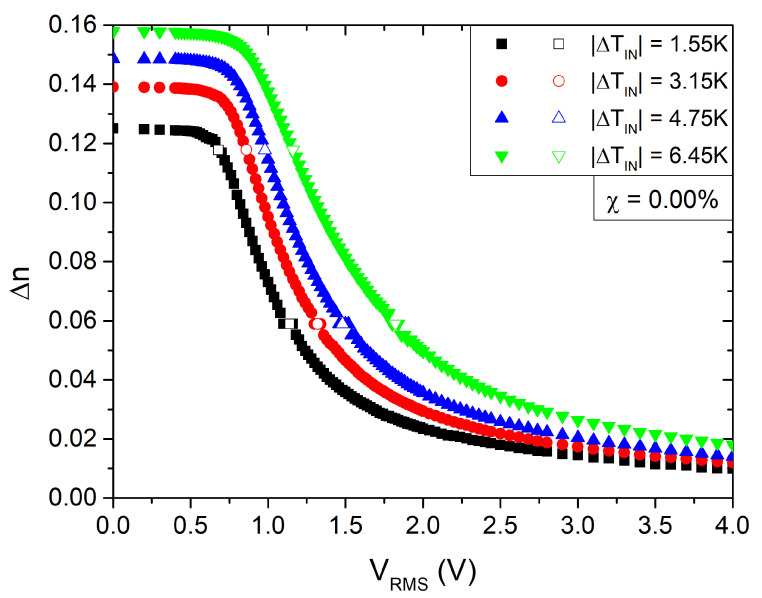
Birefringence, Δn, vs. the applied AC voltage amplitude, $V_{rms}$, calculated from the data of Figure 2.

A homogeneously aligned LC–cell was positioned within the heating-stage. The cell’s optical axis was set at an angle of ψ=±π/4 relative to the crossed polarizers (P⊥A). The intensity, *I*, of light transmitted through the cell was recorded as a function of the applied AC electric voltage across the cell at each temperature *T*. For voltages, *V*, exceeding the threshold voltage, $V_{th}$, the nematic director, n, starts to reorient towards the direction of the electric field, E, for LC–materials with a positive dielectric anisotropy Δε. This reorientation of n results in a change in the phase difference, ϕ=2πΔnd/λ, between the ordinary and extraordinary rays, since the effective birefringence, Δn, becomes a function of the field amplitude, that is, Δn(T,E). The transmitted light intensity due to the orientational transition is described by the following equation [44]

(1) $$\begin{array}{ccc} I & = & I_{0}\sin^{2}\left(2\psi \right)\sin^{2}\left(\frac{\phi }{2}\right) \end{array}$$

(2) $$\begin{array}{ccc} \phi & = & \left\{\begin{array}{cc} N\pi +2\sin^{-1}\sqrt{\frac{I}{I_{0}}} & \mathrm{for}\;N=0,2,4,\ldots \\ \left(N+1\right)\pi +2\sin^{-1}\sqrt{\frac{I}{I_{0}}} & \mathrm{for}\;N=1,3,5,\ldots \end{array}\right. \end{array}$$

where $I_{0}$ is the incident polarized light intensity. Using Equation (1), one can compute the phase change ϕ, and consequently Δn=Δn(T,χ,V) as a function of the applied AC voltage $V_{rms}$. The measurements were conducted at the frequency of 1 kHz. Odd and even values of *N* correspond to maxima and minima of the transmitted interference signal, respectively. Figure 2 depicts a typical recording of the normalized intensity $I/I_{0}$ as a function of the applied voltage at a planar cell filled with pure 8CB. Figure 3 shows the corresponding variation in birefringence. An estimation of the Fréedericksz transition threshold is usually determined by locating the cross point of the tangent to the phase slope with the level line of the phase in the absence of applied voltage. However, when a pretilt of n is present at the cell boundaries, the threshold transition is disrupted, and only an effective threshold can be defined. As we will discuss in the following sections, the presence of NPs may introduce a mean pretilt angle as well. Consequently, in order to characterize the transition, we arbitrarily define as threshold voltage the characteristic voltage at which the optical intensity changes by 10% from its value in the absence of an external field.

**Figure 4:**
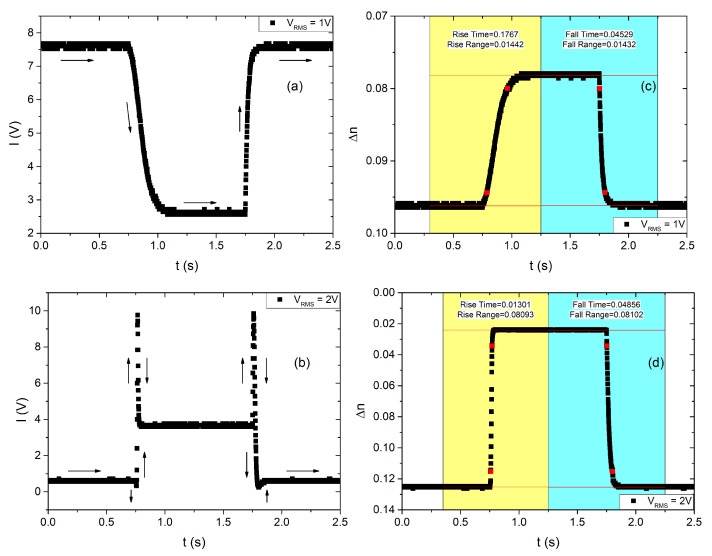
Transmitted light intensity vs. time for an electric voltage impulse of (**a**) $V_{RMS}$ = 1 V, (**b**) $V_{RMS}$ = 2 V. The corresponding birefringence variation vs. time for (**c**) $V_{RMS}=1$ V, (**d**) $V_{RMS}=2$ V.

The dynamics of the nematic director is probed by applying a pulsed AC electric voltage with a frequency of 1 kHz, amplitude ±V, and a pulse duration of 1 s, with a pulse period of 5 s. In Figure 4, the measured optical intensity (left) is presented alongside the corresponding birefringence induced by the reorientation of n (right) for cases of both low (upper part) and large (lower part) birefringence variations.

The switch-on and switch-off times are given, respectively, by the following equations

(3) $$\begin{array}{ccc} \tau_{on} & = & \frac{\tau_{off}}{V_{r}^{2}-1} \end{array}$$

(4) $$\begin{array}{ccc} \tau_{off} & = & \frac{\gamma_{1}\;d^{2}}{K_{11}\;\pi^{2}} \end{array}$$

where the ratio of the rotational viscosity, $\gamma_{1}$, to the splay elastic constant, $K_{11}$, is known as the viscoelastic coefficient, $\zeta =\gamma_{1}/K_{11}$. $V_{r}\;=\;V/V_{th}$ is the applied electric voltage in reduced units, and $V_{th}$ is the threshold voltage for Fréedericksz transition given by

(5) $$\begin{array}{c} V_{th}\;=\;\pi \;\sqrt{\frac{K_{11}}{\varepsilon_{0}\varepsilon_{a}}} \end{array}$$

where $\varepsilon_{0}$ is the vacuum permittivity and $\varepsilon_{a}$ is the dielectric anisotropy of the LC. By measuring the critical voltage of the Fréedericksz transition and the relaxation time for the reorientation of n, one can deduce the ratios $K_{11}/\varepsilon_{a}$ and $\gamma_{1}/K_{11}$. Equations (3)–(5) are strictly valid only if the anchoring is rigid. For finite anchoring energy, the above equations have to be corrected [46]. However, for strong enough anchoring, the corrections are small. In our case, the corrections for finite anchoring energy are less than 1% (see Section 5).

## 3. Experimental Results

### 3.1. Microstructure and Phase Diagrams

**Figure 5:**
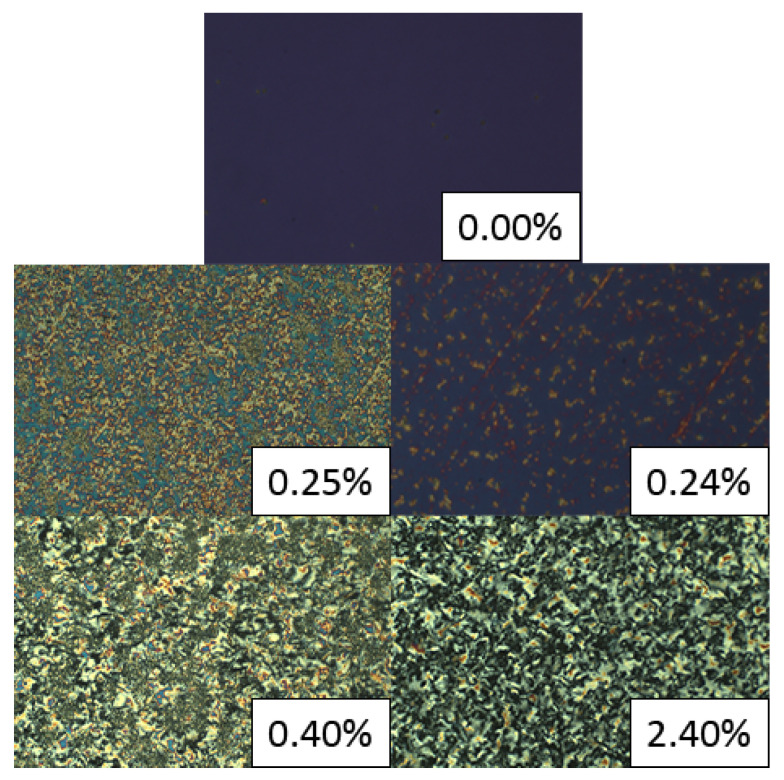
Microstructure of CdSe/ZnS QDs-8CB hybrid systems, representative cases. Tiny QDs, 0.25% and 0.40%. Large QDs, 0.24% and 2.40%. Top plate, pure 8CB. Planar cell. P⊥A.

The microstructure of the samples was observed in transmission mode under crossed polarizers in order to estimate the quality of QD dispersion in the LC-host. Figure 5 shows POM images of the samples’ microstructure for both systems. The optical axis of the samples is well defined up to a critical mass ratio $\chi_{c}\approx 0.003$ and 0.004 for the tiny and large QDs, respectively.

**Figure 6:**
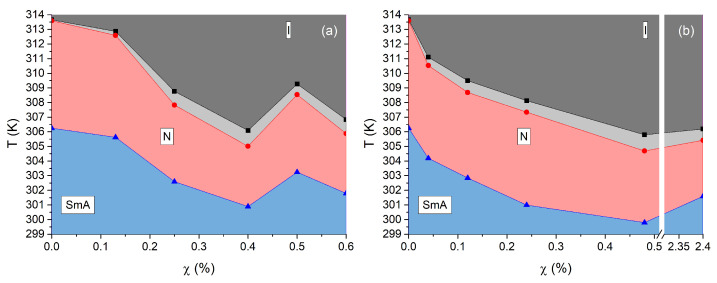
Phase diagram of the 8CB-CdSe/ZnS nanocomposites. $T_{IN}$ and $T_{NA}$ vs. the mass fraction of QDs. Tiny QDs, (**a**). Large QDs, (**b**). The nematic–isotropic coexistence range is localized between black and red points.

Figure 6 displays the phase diagrams of the two types of soft nanocomposites. The black points represent the measured isotropic–nematic (IN) phase transition temperature, $T_{IN}$, while the blue points indicate the transition temperature, $T_{NA}$, from the nematic to smectic-A (NA) phase for various χ values of the nanocomposites. The range between the black and red points corresponds to the coexistence range of isotropic and nematic phases. The lines in the graph serve as visual guides. All temperatures were measured while cooling from the isotropic phase at a rate of 0.1$\frac{K}{\min}$. $T_{IN}$ and $T_{NA}$ decrease with increasing χ up to approximately χ≈ 0.4–0.5%. Specifically, at χ≈0.4%, $T_{IN}$ and $T_{NA}$ decrease by 7.5 K and 5.3 K, respectively, for t-type systems, and by 7.8 K and 6.4 K, respectively, for *ℓ*-type systems. The temperature range of the nematic phase, denoted by $\Delta W_{N}$, also reduces with increasing χ, by 2.2 K for t-type and by 1.4 K for *ℓ*-type systems. That is, the absolute shift of the clearing temperature, $\Delta T_{IN}$, increases monotonically with χ up to a maximum of 7.5 K (7.8 K) at χ∼0.4% for tiny QDs (large QDs). For χ>0.4%, aggregation becomes significant. Consequently, there is a reduction in the effective number of NPs, and open structures emerge in the nanocomposites, which we hereafter refer to as NP-networks. Note that before the appearance of NP-networks, the samples undergo the IN transition at the same temperature in their entire volume, that is, the NPs are relatively well-dispersed in a homogeneous manner.

### 3.2. Orientational Order

**Figure 7:**
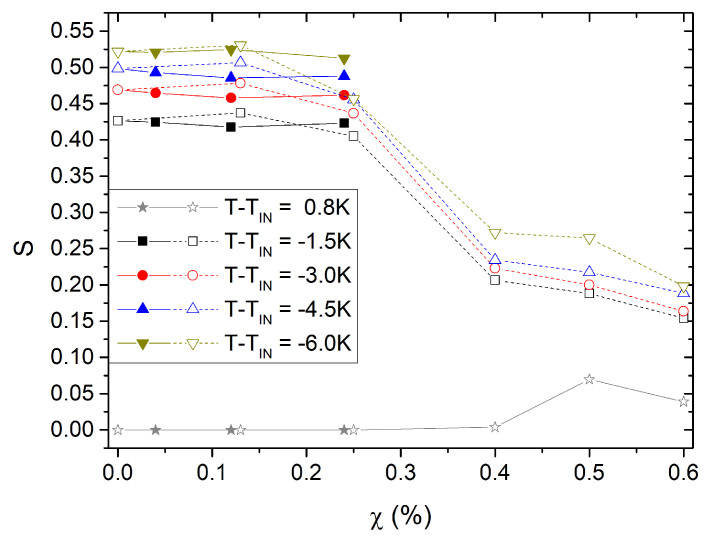
Isotherms of the orientational order parameter, S(T,χ), as a function of mass fraction for the 8CB-QDs nanocomposites. Open symbols, tiny QDs. Solid symbols, large QDs.

Figure 7 shows several isotherms of the orientational order parameter, *S*, as a function of the mass ratio, χ, for both diameters of the QDs. The behavior of *S* in the nematic phase exhibits a similar pattern as in pure 8CB, that is, the orientational order continuously increases as the nematic phase cools. However, for $\chi \geq \chi_{c}$, the orientational order saturates towards values S∼0.25 below the value of orientational order for the pure material at its clearing temperature $S_{NI}^{0}\approx 0.35$. Note also that the isotherms show first an increase of *S* with concentration for χ∼0.001 followed by a decrease for higher concentrations. This measured nematic order reduction that arrives for the nematic crystal is constrained elastically by the presence of the NPs, resulting in a local reorientation of the nematic director. This strain field induces a severe order reduction above some threshold $\chi_{c}$ value in the range 0.003<χ<0.004. Increasing χ results in a phase separation with the establishment of NP-networks that trap LC and a continuum phase with lower NP concentration, where the order parameter is practically not sensible to the temperature variation, and its amplitude is saturated at values of *S* lower than ∼0.35. It is worth noting that isotherms of samples with NP-networks show orientational order even above the clearing temperature of the bulk LC; see isotherm at $T-T_{IN}=0.8$ K in Figure 7.

### 3.3. Fréedericksz Transition Threshold

**Figure 8:**
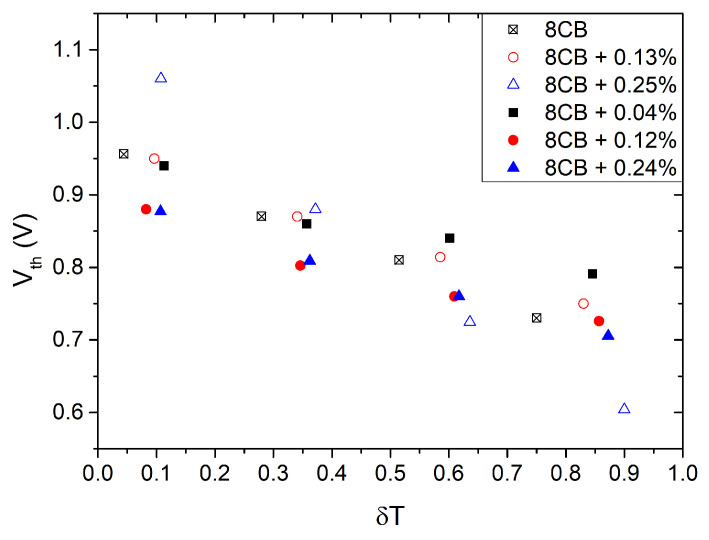
Iso-χ of $V_{th}$ vs. $\delta T=\left(T-T_{NA}\right)/\left(T_{IN}-T_{NA}\right)$. Open symbols, t-system. Solid symbols, *ℓ*-system. Method 10%.

Figure 8 shows the threshold voltage of the Fréedericksz transition as a function of the reduced temperature ($\delta T=\left(T-T_{NA}\right)/\left(T_{IN}-T_{NA}\right)$) at various concentrations of QDs. The left part of the figure pertains to tiny QDs and the right part pertains to large ones. In all cases, $V_{th}$ increases as the nematic phase cools. For the large QDs, at χ=0.004, the threshold $V_{th}\left(\chi =0.004\right)$ is higher than $V_{th}^{0}$ near the clearing temperature and $V_{th}\left(\chi =0.004\right)$ becomes nearly equal to $V_{th}^{0}$ deep in the nematic phase. However, for higher χ values, the threshold becomes smaller than $V_{th}^{0}$ by up to 8%. For the tiny QDs, at χ=0.0013, the threshold is the same as for pure 8CB. However, at χ=0.0025, the threshold exhibits significant temperature dependence. Close to $T_{IN}$, we measure a 20% lower $V_{th}$ than $V_{th}^{0}$ while close to $T_{NA}$, $V_{th}\left(0.0025\right)>V_{th}^{0}$. For samples with χ≥0.004, no Fréedericksz transition is observed. Instead, only a slow reduction of the signal with increasing voltage is detected.

### 3.4. Switch-On and Switch-Off Times

**Figure 9:**
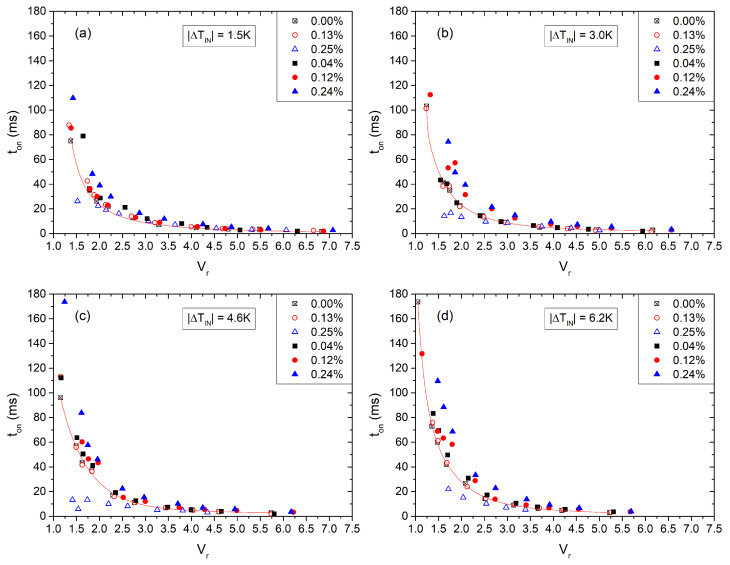
Switch-on time of 8CB-QDs nanocomposites for several χ-values as a function of the applied voltage at $|\Delta T_{IN}|=T_{IN}-T=1.5,\;3.0,\;4.6,\;6.2$ K, subfigures (**a**–**d**) respectively. Open symbols, tiny QDs. Solid symbols, large QDs.

Figure 9 displays iso-χ curves of the measured rise time, $\tau_{on}$, of the nanocomposites’ response to a voltage impulse, as a function of the voltage rms amplitude. The rise time is defined as the transit time for the light intensity increase from 10% to 90%. Each sub-figure presents the acquired data at a constant temperature. Temperature measurements are relative to the clearing temperature $|\Delta T_{IN}|=T_{IN}-T=1.5,\;3,\;4.6,\;6.2$ K. Voltage values are presented in reduced units ($V_{r}$). For applied voltages higher than $V_{r}>3$, we observe that all $\tau_{on}\left(\chi \right)$ converge towards $\tau_{{}_{on}}^{0}=\tau_{{}_{on}}\left(\chi =0\right)$ (pure 8CB), which corresponds to the behavior of pure 8CB and has a timescale of approximately 1 ms. However, for smaller $V_{r}$ values, deviations from $\tau_{{}_{on}}^{0}$ become apparent. Notably, these deviations are particularly significant at χ∼0.25% for both sizes of QDs. In particular, for the large QDs, $\tau_{on}$ is larger than $\tau_{{}_{on}}^{0}$ and increases with concentration. In contrast, for the tiny QDs, $\tau_{on}$ remains nearly the same as $\tau_{{}_{on}}^{0}$ for χ=0.0013 and becomes faster for χ=0.0025.

**Figure 10:**
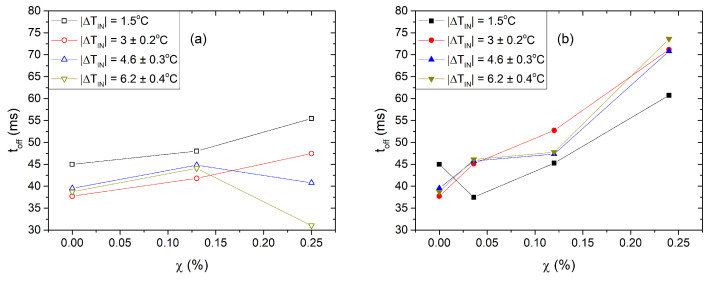
Switch-off time, $\tau_{off}$, vs. the mass fraction χ of the 8CB-QDs nanocomposites. Isotherms at $|\Delta T_{IN}|=T_{IN}-T=1.5,\;3.0,\;4.6,\;6.2$ K. Tiny QDs, (**a**). Large QDs, (**b**). The error in the temperatures is referred to the scattering of the temperature from one sample to another. The resolution of T is 0.1 K.

The decay time is defined as the transit time for the light intensity decrease from 90% to 10%. Figure 10 shows isotherms of $\tau_{off}$ as a function of χ, with tiny QDs on the left and large QDs on the right. One observes that $\tau_{off}$ significantly increases with χ for the system with large QDs. In particular, $\tau_{off}$ roughly doubles its value compared to that of pure 8CB. For nanocomposites containing tiny QDs, $\tau_{off}$ exhibits a mild variation on χ with respect to $\tau_{off}^{0}$, for the isotherms at $|\Delta T_{IN}|=1.5,3.0$ K. Note also that, when approaching the SmA phase, $|\Delta T_{IN}|=4.6,6.2$ K and at χ≈0.0025, $\tau_{off}$ begins to decrease.

**Figure 11:**
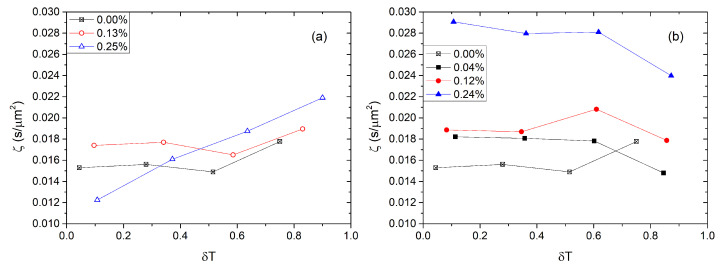
Viscoelastic coefficient of the 8CB–QDs nanocomposites as a function of the reduced temperature δT. (**a**) Open symbols, t-type nanocomposites, iso-χ for 0.13% and 0.25%. (**b**) Solid symbols, *ℓ*-type nanocomposites, iso-χ for 0.04%, 0.12%, and 0.24%.

The decay time following the removal of the field is proportional to the viscoelastic coefficient, $\tau_{off}∼\zeta =\gamma_{1}/K_{11}$. In Figure 11, we plot iso-χ curves of the viscoelastic coefficient, ζ, as a function of temperature. In the case of pure 8CB, on heating $\zeta \left(\chi =0,T\right)=\zeta_{0}\left(T\right)$ initially decreases and then increases, approaching the clearing temperature, passing though a minimum, which is a well-known behavior. For nanocomposites containing large QDs, the viscoelastic coefficient is slightly higher than that of pure 8CB ($\zeta_{0}$) when χ<0.2%. However, at χ=0.25% and a few degrees below $T_{IN}$, ζ doubles in value ($\zeta /\zeta_{0}\approx 2$). For nanocomposites with tiny QDs, ζ increases up to 10% compared to $\zeta_{0}$. Remarkably, at χ=0.0025, ζ exhibits a monotonic variation with *T*, that is, it continues to decrease by cooling deep in the nematic phase.

## 4. Anchoring

The form of the experimental I(V) curves for pure 8CB (Figure 2 and Figure 3) reveals a finite anchoring condition and a small pretilt angle. One can estimate the polar anchoring energy, $w_{p}$, at the cell–LC interfaces by using the high electric field method [47,48,49,50] based on the linear dependence of the birefringence as a function of the inverse voltage [47]. We measured a saturation field $E_{b}\approx 17.3\;V/$μm. In a first approximation, the polar anchoring energy strength $w_{p}$ was calculated by equating the electric field energy with the interfacial layer energy, which resulted in $w_{p}\;=\;2.4\times 10^{-4}\;J/m^{2}$. For the numerical calculations, we used the values $\varepsilon_{a}=7$, K=3pN[29] and the dielectric permittivity of vacuum $\varepsilon_{0}=8.85\times 10^{-12}\;F/m$. Subsequently, the extrapolation length is $b=K/w_{p}\approx 13\;\mathrm{nm}$. One can easily verify that the corrections for $V_{th}$ and the switching times are smaller than 1%, and, therefore, we neglect in our analysis finite anchoring effects.

Finally, we calculated the extrapolation length at the surface of the QDs for both systems by following the main argument of [41]. At equilibrium, one can expect that the surface energy at the NP–LC interface for a homogeneous nematic is of the same order as the distortion elastic energy. This condition can be expressed as

(6) $$\begin{array}{c} W_{d}∼\frac{K}{\xi_{d}^{2}}d_{np}^{3} \end{array}$$

where $d_{np}$ represents the mean distance between NPs in the nanocomposite, $\xi_{d}$ stands for the typical elastic distortion length of the nematic phase around the NP, and *K* denotes an effective elastic constant. At the critical volume fraction, the elastic distortion length is taken of the same order as the mean distance between NPs, $\xi_{d}∼d_{np}$. Consequently, Equation (6) yields $W_{d}∼Kd_{np}$. Finally, one finds

(7) $$\begin{array}{c} \phi_{c}=\frac{1}{6\pi^{2}}{\left(\frac{b}{D}\right)}^{3} \end{array}$$

where $\phi \approx \chi \rho_{lc}/\rho_{qd}$ is the volume fraction of the NPs, $\rho_{lc}$ is the LC density and $\rho_{qd}$ is the density of the QDs (including the surface layer). For the tiny QDs, we find b∼2 nm, and for the large QDs, b∼3.7 nm. This result implies that the homeotropic anchoring on the NP–LC interface is stronger for tiny QDs compared to large QDs.

Using these *b* values one can calculate the anchoring energy at the NP–LC interface

(8) $$\begin{array}{c} w_{qd}=\frac{K}{b}\Rightarrow \left\{\begin{array}{c} w_{qd}^{t}=1.4\times 10^{-3}\;J/m^{2}\\ w_{qd}^{\ell }=0.8\times 10^{-3}\;J/m^{2} \end{array}\right. \end{array}$$

that is, the anchoring energy on the NP–LC interface is stronger than the anchoring energy on the cell–LC interface.

## 5. Discussion and Conclusions

The phase diagrams (Figure 6) of both systems reveal that the clearing temperature is suppressed with an increase of the NPs’ mass fraction, following a non-linear law. In molecular mixtures $T_{IN}$ typically follows a linear law. A similar linear behavior is usually observed in nanocomposites as well. Phenomenologically, the linearity is derived from a coupling term, often of the form $∼\phi S^{2}$, in the Landau–de Gennes energy expansion. Note that, for low enough values of the mass fraction χ, the volume fraction ϕ is proportional to χ. This term primarily describes the dilution effect in LC by isotropic particles, which lead to a decrease in the mean intermolecular interaction strength, resulting in a reduction of the clearing temperature. From the observed non-linearity in our systems, it is inferred that higher order coupling terms are important, that is, a quadric $∼\phi^{2}S^{2}$ term is necessary in the case of the *ℓ*-type system to describe the parabolic form of the transition line. For the t-type system, one observes a change of the curvature sign indicating that even higher order, $∼\phi^{3}S^{2}$, terms are necessary to describe the data. However, one should be cautious, and, in order to infer conclusions, more data should be acquired. Note also that data above $\chi_{c}$ concern a phase-separated system. In addition, when comparing the phase diagrams of the two systems, one can observe that the initial $T_{IN}$ depression at χ≈0.1% is much deeper, about 5 K, for the *ℓ*-type system than for the t-type one, about 1 K.

Our experimental results demonstrate that, in dilute nanocomposites, the NPs induce disorder, highlighting the sensitivity of orientational order even to weak perturbations. This effect presents a threshold above which the nematic phase is disrupted. Our current results on the disorder induced by NPs align with previous experimental observations in other systems [41] and a previous investigation in *ℓ*-type systems [43]. Moreover, they are consistent with theoretical predictions [42]. QDs introduce deformations in the nematic director field due to their interaction with the mesogenic molecules. In particular, the homeotropic alignment at their interface with the LC is incompatible with the homogeneous (planar) alignment induced by the cell’s interfaces with the LC. As is well known, this incompatibility can give rise to topological defects depending on the size of the QDs and the extrapolation length [22], as well as elastic strains in the LC matrix [41]. At high enough concentrations, the QDs interact with each other through the elastic distortion of the orientational field, and in some cases, translational order emerges, forming colloidal crystals [43]. Since the distortion of the nematic field induces variations in the tensorial order parameter [42], it consequently leads to changes in the properties of the nematic host.

At this point, a remark regarding the critical mass ratio is in order. In the limits of our experimental resolution, the experimental data show that the critical mass for the two sizes of QDs is different. Specifically, for the t-type system, the critical mass fraction is approximately 25% lower than that for the *ℓ*-type system. For the same mass fraction, the concentration and total surface area are much higher/larger for the tiny QDs. However, the effect is not solely dependent on concentration; it also relies on other factors such as anchoring strength at the QDs–LC interface and nano-aggregation, which reduces the effective concentration. Further experiments are needed to quantitatively study this dependence.

In order to estimate the variation of $\zeta =\gamma_{1}/K$ with *S*, one usually assumes that $K∼S^{2}$ and that the rotational viscosity varies with the order parameter following a power law $S^{x}$, where 0≥x≥2[51]. That is, $\zeta ∼S^{x-2}$. For pristine LCs, ζ increases close enough to $T_{IN}$, meaning that x<2. For t-QDs, ζ shows the same trend as in pure system close to $T_{IN}$, that is, viscosity decreases slower than $S^{2}$. For *ℓ*-QDs, ζ(χ≠0) decreases on heating towards $T_{IN}$, that is, viscosity decreases faster than $S^{2}$, i.e., x>2. This result seems genuine and demands more experimental investigations. We also note that, for the t-QDs system with χ=0.0025, the rotational viscosity of the nematic phase keeps decreasing on cooling from $T_{IN}$, even approaching the smectic-A phase where normally $\gamma_{1}$ should diverge. This result could be suggesting that the transition becomes slightly first-order at large concentration of NP. Nonetheless, we note that the system is yet half a degree above $T_{NA}$ and it is probable that pretransitional effects are not strong enough yet. Further experimental investigations are necessary in order to conclude this case.

In conclusion, we doped QDs of two different diameters in 8CB and constructed the phase diagram of the anisotropic soft-nanocomposites. From these phase diagrams, we concluded that both the nematic and the smectic-A phase of the 8CB are preserved in presence of the QDs. Both transition temperatures are depressed with increasing concentration of the QDs and the nematic phase temperature span tapers by ≈20%. The clearing temperature’s drop is non-linear with the concentration in opposition to the case of molecular mixtures and very dilute nanocomposites. Nevertheless, the $T_{IN}$ shift is monotonous at least up to concentrations where NP-networks appear. Electrooptical measurements on threshold voltage of the Fréedericksz transition, $V_{th}$, and switching times show that, in the investigated range of concentrations, NPs may strongly affect electrooptical properties of the LC. $V_{th}$ varies mildly with the QDs concentration. The response of the nanocomposites with tiny QDs becomes faster, while those with large QDs become slower compared to the response of the pure system within the investigated range of sizes. These deviations become stronger when temperature reduces. The viscoelastic coefficient, ζ, in the nematic phase of the nanocomposites depends on the NPs’ size in an essential way. More precisely, we found that, for large QDs, ζ strongly increases with the concentration of the nanocomposites up to ($\zeta \approx 2\zeta_{0}$). Moreover, its behavior qualitatively changes close to $T_{IN}$, that is, ζ continues to decrease upon approaching the clearing temperature. Tiny QDs mildly impact the system, which qualitatively behaves as the pure 8CB system. Globally, t-QDs show a smaller ζ than *ℓ*-QDs. Finally, we calculated the anchoring energy at the surface of the QDs, which seems to depend upon the diameter of the QDs. Based on the results presented above, it is intriguing to explore size effects across a broader range and in other systems. 

## Data Availability

The research data of the paper are available on demand.
